# Topical NSAIDs, intravitreal dexamethasone and peribulbar triamcinolone for pseudophakic macular edema

**DOI:** 10.1186/s12886-021-02132-w

**Published:** 2021-11-05

**Authors:** Javier Obis, Luis Arias, Daniel Lorenzo, Noel Padron-Perez, Pere Garcia-Bru, Estefania Cobos, Rahul Morwani, Jose Caminal

**Affiliations:** Ophthalmology department, Bellvitge University Hospital, Carrer de la Feixa Llarga, s/n, 08907. Hospitalet de Llobregat, Barcelona, Spain

**Keywords:** Pseudophakic macular edema / Irvine Gass syndrome, Topical NSAIDs, Intravitreal dexamethasone, Peribulbar triamcinolone, Central retinal thickness, Central choroidal thickness

## Abstract

**Background:**

The purpose of this study is to assess the effectiveness of topical nonsteroidal anti-inflammatory drugs (NSAIDs) and corticosteroids (intravitreal dexamethasone and peribulbar triamcinolone) in treating pseudophakic macular edema (PME).

**Methods:**

Retrospective study of 33 eyes. Variables included best corrected visual acuity (BCVA; logMAR scale) and central retinal thickness (CRT) and central choroidal thickness (CCT) assessed with swept-source OCT. All patients were initially prescribed topical NSAIDs and reevaluated after 2 months. If improvement in BCVA or CRT was noted, topical NSAIDs were continued until resolution. If no improvement was observed at 2 months or subsequent visits, intravitreal dexamethasone implant was performed. Patients who refused intravitreal treatment were offered peribulbar triamcinolone.

**Results:**

After treatment with topical NSAIDs for a median of 2 months, BCVA increased significantly from 0.5 to 0.3 while CRT decreased significantly from 435 to 316 μm. PME resolved in 19 of the 33 eyes (57.6%). Of the 14 recalcitrant cases, 13 were treated with corticosteroids. Of these 13 cases, 9 (69.2%) resolved. BCVA increased non-significantly from 0.7 to 0.4. CRT and CCT decreased significantly from 492 to 317 μm and from 204 to 182 μm respectively.

**Conclusions:**

The overall success rate of the treatment algorithm was greater than 80%, a remarkable finding considering that no randomized study has yet been conducted to determine the optimal therapeutic protocol for PME. This is the first study to evaluate choroidal thickness in PME using SS-OCT, which could play a key role in its pathophysiology and provide useful information to improve the management of PME.

## Background

The term postsurgical macular edema is used to describe the macular edema that appears in some eyes after ocular surgery such as cataract surgery or vitrectomy, and it is an important cause of postsurgical vision loss. Macular edema that develops after complicated or uncomplicated cataract surgery is known as pseudophakic macular edema (PME) or Irvine-Gass syndrome (IGS). PME was first described by Irvine in 1953 [1] and subsequently documented on angiography by Gass and Norton in 1966 [2].

The reported incidence of PME detected with optical coherence tomography (OCT) ranges from 5.5 to 9% [3, 4]. The incidence of clinically-significant PME, which includes decreased visual acuity or metamorphopsia, ranges from 0.95 to 2.35% [5, 6]. PME typically develops from 4 to 12 weeks after the surgery, with a peak incidence around week 6 [7]. Several risk factors have been suggested, including intraoperative capsular rupture, epiretinal membrane, diabetes or uveitis [4, 6, 7]. Although the pathophysiology of PME is not well-understood, some reports suggest that it may be related to an inflammatory process that disrupts the blood-retina barrier, leading to increased vascular permeability and subsequent fluid accumulation [7, 8].

Although several different treatment options for PME have been investigated in recent years [9, 10], no randomized studies have been conducted to determine the optimal therapeutic protocol for PME. In routine clinical practice, topical nonsteroidal anti-inflammatory drugs (NSAIDs) are commonly used as first-line therapy, either alone or in combination with oral acetazolamide [9, 10]. If first-line treatment is unsuccessful, a wide range of second-line treatments are available [11], most commonly corticosteroids such as subtenon or peribulbar triamcinolone [12] or intravitreal dexamethasone [11, 13–16]. Other second-line alternatives have been proposed, including intravitreal anti-VEGF injections (with conflicting results) [17, 18], or infliximab (which may be associated with retinal toxicity) [19, 20]. Treatment selection is further complicated by the fact that PME (defined as macular edema after cataract surgery) and macular edema occurring after vitrectomy could be two different entities that respond differently to treatment [21]. Considering the aforementioned, more randomized controlled studies with larger number of patients would be necessary to evaluate the treatment of clinically significant PME.

In this context, the aim of the present study was to retrospectively assess the effectiveness of topical NSAIDs, intravitreal dexamethasone and peribulbar triamcinolone in patients with clinically-significant PME treated in a real-world clinical setting. In this case series, we evaluated best-corrected visual acuity (BCVA) and central retinal and choroidal thickness using swept-source OCT (SS-OCT). Finally, we describe a proposed treatment algorithm.

## Material and methods

This was an observational, retrospective, consecutive case series involving patients treated at a tertiary care hospital (Bellvitge University Hospital in Barcelona, Spain). All the data were obtained from the medical records of patients with PME who had been previously treated following the treatment algorithm that will be explained below. Inclusion criteria were as follows: 1) cataract surgery (complicated or uncomplicated), 2) presence of symptomatic postoperative macular edema on SS-OCT (in all cases, visual acuity improved after cataract surgery but worsened afterwards). Exclusion criteria included a history of any of the following: diabetic retinopathy including diabetic macular edema, retinal vein occlusion, posterior uveitis, age-related macular disease (including presence of drusen), central serous chorioretinopathy, or any other condition that could potentially confound the diagnosis, affect retinal or choroidal thickness, or modify treatment response. In order to rule out these conditions, no patient with history of diabetes was included in this study, and all the patients underwent slit lamp examination, funduscopy and SS-OCT examination before cataract surgery.

BCVA was evaluated (logMAR scale) and SS-OCT was performed to assess central retinal thickness (CRT) and central choroidal thickness (CCT) at diagnosis of PME and thereafter every 2 months.

The Deep Range Imaging (DRI) Triton SS-OCT device (Topcon, Tokyo, Japan) was used to measure CRT and CCT, and to determine the presence of intraretinal and/or subretinal fluid. The DRI Triton SS-OCT utilizes a 1050 nm wavelength light, with 100,000 A-scans per second, and axial and transverse resolutions of 8 and 20 μm, respectively. We used the Radial protocol, which consists of a 12 mm radial scan of the macular area to obtain thickness measurements of the nine macular areas of the Early Treatment Diabetic Retinopathy Study (ETDRS). The retinal and choroidal values of the central ETDRS area were registered and included in our study. The DRI Triton SS-OCT yields a quality scale that ranges from 0 (lowest quality) to 100 (highest quality). We included only images whose score was > 50.

The following clinical and demographic variables were registered: patient sex and age; duration of topical NSAIDs; number of corticosteroids injections; and time elapsed from surgery to diagnosis of PME.

**Fig. 1:**
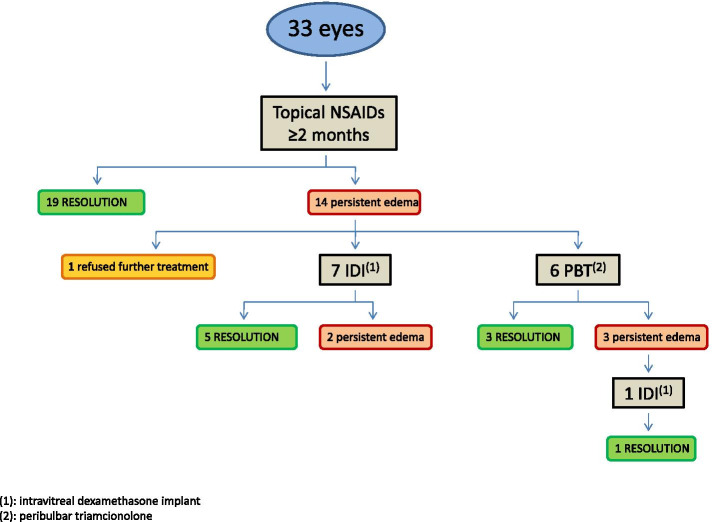
Treatment algorithm and flow chart. The number of patients who received each treatment and the number of cases in which the edema resolved or persisted are shown

Figure 1 shows the treatment algorithm applied, the number of patients who received each treatment, and the number of cases in which the edema resolved or persisted. In all cases, first-line treatment consisted of once-daily topical NSAIDs (nepafenac 3 mg/ml; Nevanac, Novartis) administered for 2 months, after which the BCVA was evaluated, and the CCT and CRT were measured by SS-OCT.

In patients who showed improvement in BCVA or CRT decrease at the first follow-up visit (2 months after starting topical NSAIDs), topical NSAIDs were continued and a second follow-up was scheduled for 2 months later. At that second visit, if the edema had resolved (no intraretinal or subretinal fluid on SS-OCT), NSAIDs were discontinued and the patient was scheduled for another follow-up visit 2 months later to verify the continued absence of the edema. If at any subsequent visit one patient showed improvement in BCVA or CRT decrease but the edema had not resolved completely, topical NSAIDs were continued and the patient was scheduled for another follow-up visit. Thus, topical NSAIDs were continued until complete resolution of the edema or until no response was observed at a follow-up visit.

At the first follow-up visit, patients who showed no response to the two-month course of topical NSAIDs (no improvement in BCVA and no decrease in CRT) were prescribed an intravitreal dexamethasone implant (Ozurdex, Allergan). Patients who exhibited an initial improvement in BCVA or CRT with topical NSAIDs at any follow-up visit but showed no improvement at a subsequent visit, were also prescribed intravitreal implant of dexamethasone.

After dexamethasone implant, follow-up visits were scheduled every 2 months. In patients in whom the edema decreased but failed to resolve completely (persistence of intraretinal or subretinal fluid on SS-OCT) at least 4 months after the first implant, a second dexamethasone implant was performed. In contrast, patients whose edema resolved (no intraretinal or subretinal fluid on SS-OCT) after the dexamethasone implant were followed for at least 6 months to verify no recurrence of the edema. Dexamethasone implants were discontinued when persistent absence of edema was verified (healed PME) or when no decrease of intraretinal or subretinal fluid was observed at all in the follow-up visits (refractory PME). Finally, patients who refused intravitreal treatment were offered peribulbar triamcinolone (Trigon, Bristol-Myers Squibb).

Written informed consent was deemed unnecessary as this was a retrospective study carried out in the context of routine clinical practice. All patient data were anonymized for this study. All confidential data are protected according national legislation. This study and manuscript have been approved by the Research Ethics Committee of Bellvitge University Hospital.

### Statistical analysis

All variables were registered in a spreadsheet (Microsoft Excel). Statistical analysis was performed using the SPSS software program, v. 20.0 (SPSS, Inc., Chicago, IL). All variables showed a non-normal distribution, except for age (Shapiro-Wilk test). Consequently, the Wilcoxon test was used to compare the medians of the variables. Differences were considered statistically significant for *p* ≤ 0.05.

## Results

Thirty-three eyes from 33 patients were included in the study. Table 1 shows baseline parameters. Mean patient age was 73.8 years (SD ±9.27). Twenty-three patients (69.7%) were male. Of the 33 patients, 48.5% (*n* = 16) were consequent to uncomplicated cataract surgery, and 51.5% (*n* = 17) were consequent to complicated cataract surgery (posterior capsular rupture). The median time to diagnosis was 1 month. Mean follow-up was 9 months.

**Table 1:** General characteristics of the sample

Number of eyes	33
**Mean age, years (range)**	73.8 (53–93)
**Sex, male (%)**	23 (69.7%)
**Cataract surgery, complicated (%)**	17 (51.5%)
**Time to diagnosis, months (range)**	1 ( [1-]8)
**Follow-up time, months (range)**	9 ( [4-]29)
**Baseline BCVA, logMAR**	0.5 (0 [2-].1.30)
**Baseline CRT,** μm	435 (27 [2-]786)
**Baseline CCT, μm**	253 (79–358)

*Abbreviations*: *BCVA* Best corrected visual acuity, *logMAR* Logarithm of the minimum angle of resolution, *CRT* Central retinal thickness, *CCT* Central choroidal thickness

Median baseline values were as follows: BCVA, 0.5 logMAR range 0 [2-].1.30 (decimal scale 0.32 range 0.63–0.05); CRT, 435 μm range 27 [2-]786 μm; and CCT, 253 μm range 79–358 μm. There were no significant differences in baseline parameters between patients who had undergone complicated vs. uncomplicated cataract surgery.

Table 2 shows the baseline and post-treatment (topical NSAIDs) results. Median BCVA improved to 0.3 (decimal scale 0.5); median CRT decreased to 316 μm; and median CCT decreased to 256 μm. Compared with the baseline values, significant differences (Wilcoxon signed-rank test) were observed for BCVA (− 2.914, *P* = 0.004) and CRT (− 2.963, *P* = 0.003), but not for CCT (− 1.357, *P* = 0.175).

**Table 2:** Characteristics of the 33 patients treated with NSAIDs

	Baseline	Final	P*
BCVA, logMAR (range)	**0.5** (0.2–1.30)	**0.3** (0.0–1.30)	**0.004**
CRT, μm (range)	**435** (272–786)	**316** (227–871)	**0.003**
CCT, μm (range)	253 (79–358)	256 (76–382)	0.175
Resolution of PME, n (%)	19/33 (**57.6%**)		
Duration of treatment, months (range)	2 (2–9)		

* Wilcoxon signed-rank test

*Abbreviations*: *NSAIDs* Non-steroidal anti-inflammatory drugs, *logMAR* Logarithm of the minimum angle of resolution, *CRT* Central retinal thickness, *CCT* Central choroidal thickness, *PME* Pseudophakic macular edema

In 19 eyes (57.6%) the edema resolved with topical NSAIDs after a median of 2 months. In the 14 eyes (42.4%) that failed to respond to topical NSAIDs, second-line corticosteroid treatment was proposed; however, one patient refused any further treatment and thus 13 patients (39.4%) received second-line therapy. Of these, seven received intravitreal dexamethasone implant, which resolved the edema in five cases (71.4%) after a median of 2 injections. The remaining six patients received peribulbar triamcinolone (because they preferred to avoid intravitreal injection), which resolved the edema in three cases (50%) after a median of one injection. One of the patients with persistent macular edema after peribulbar triamcinolone agreed to receive intravitreal dexamethasone, which resolved the edema after two injections. (Fig. 1)

Given the limited number of patients who received intravitreal dexamethasone implant and/or peribulbar triamcinolone (*n* = 13), we decided to group these patients into a single group (corticosteroid) for statistical analysis. Table 3 shows the baseline and post-treatment results. Nine of the 13 cases (69.2%) resolved with second-line corticosteroid treatment. Median BCVA improved from 0.7 to 0.4 (decimal scale from 0.2 to 0.4), but the difference was not statistically significant (Wilcoxon signed-rank test − 1.577, *P* = 0.115); median CRT decreased significantly from 492 to 317 μm (− 3.036, *P* = 0.002); and median CCT decreased significantly from 204 to 182 μm (− 3.185, *P* = 0.001).

**Table 3:** Characteristics of the 13 eyes treated with corticosteroids (IDI + PBT)

	Baseline	Final	P*
BCVA, logMAR (range)	0.7 (0. 30-1.30)	0.4 (0.05–1.30)	0.115
CRT, μm (range)	**492** (359–871)	**317** (202–625)	**0.002**
CCT, μm (range)	**204** (76–382)	**182** (66–284)	**0.001**
Resolution of PME	9/13 (**69.2%**)		
Number of injections	IDI: 2 (1-5)	PBT: 1 (1-2)	

* Wilcoxon signed-rank test

*Abbreviations*: *IDI* Intravitreal dexamethasone implant, *PBT* Peribulbar triamcinolone, *logMAR* Logarithm of the minimum angle of resolution, *CRT* Central retinal thickness, *CCT* Central choroidal thickness, *PME* Pseudophakic macular edema

**Fig. 2:**
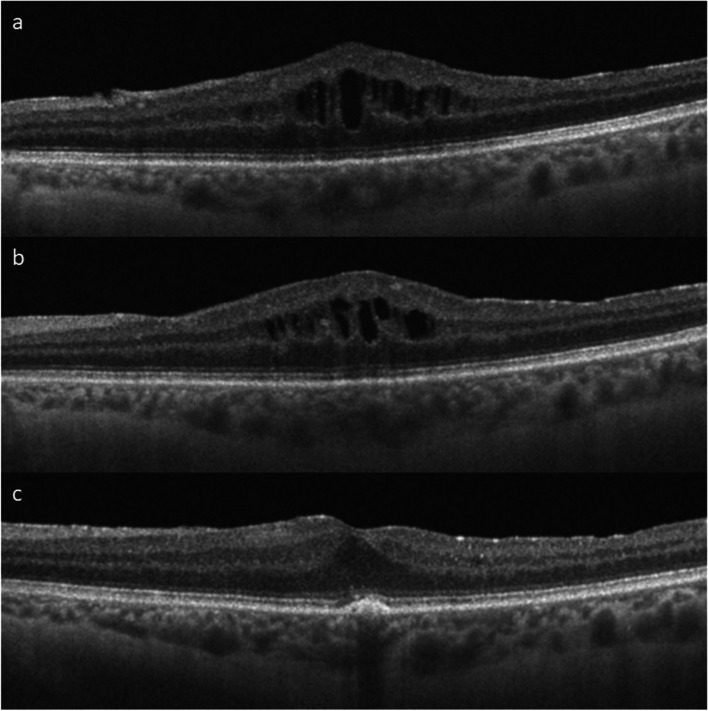
SS-OCT scans of a patient who received intravitreal dexamethasone implant (IDI) after suboptimal response to topical NSAIDs. **a** Baseline scan. BCVA: 0.6 logMAR, CRT: 483 μm, CCT: 267 μm. **b** Scan after 2 months of topical NSAIDs. BCVA remained unchanged (0.6 logMAR), CRT decreased to 471 μm, CCT was 269 μm. Since BCVA had not improved and CRT had improved scarcely and macular edema persisted, IDI was administered. **c** SS-OCT scan at 12 months post-IDI. BCVA improved to 0.4 logMAR, CRT decreased to 335 μm, macular edema was resolved, and CCT decreased to 227 μm. Disruption of external retinal layers can be observed. No drusen were found in funduscopy

Figure 2 shows the SS-OCT scans of a patient who required intravitreal dexamethasone after suboptimal response to topical NSAIDs.

No adverse treatment-related events were observed (keratitis or other ocular surface disturbances with NSAIDs; significant intraocular pressure elevation with corticosteroids; endophthalmitis with intravitreal dexamethasone implant).

## Discussion

The present retrospective study was performed to evaluate the effectiveness of topical NSAIDs and corticosteroids for the treatment of pseudophakic macular edema in real-life clinical practice. In over 80% of the patients, the macular edema was successfully resolved by following the treatment algorithm described in Fig. 1. Of the 33 cases, 19 (57.6%) resolved (no intraretinal or subretinal fluid) with topical NSAIDs after 2 to 9 months of treatment. Median BCVA improved significantly from 0.5 to 0.3 and CRT decreased significantly from 435 to 316 μm. Of the 13 eyes that received second-line corticosteroid treatment, the edema resolved in 9 eyes (69.2%), with a non-significant improvement in median BCVA (from 0.7 to 0.4), and a significant decrease in CRT (from 492 to 317 μm) and CCT (from 204 to 182 μm). Intravitreal dexamethasone implant was effective even in a case that had not responded previously to topical NSAIDs and peribulbar triamcinolone.

Guclu et al. [22] applied a 3 month course of topical nepafenac 0.1% four times a day in 30 eyes with PME, reporting a significant improvement in ETDRS BCVA from 20.9 to 32.9 letters (logMAR scale: approximately 1.3 to 1.1) and a significant decrease in CRT from 501.2 to 364.9 μm after 6 months. However, the resolution rate of macular edema was not reported.

Yüksel et al. [23] applied a 12 week course of topical nepafenac 0.1% three times a day in 24 eyes with PME, reporting a significant logMAR BCVA improvement from 0.84 to 0.37, and a significant decrease in CRT from 483.7 to 278.0 μm after 6 months. The edema resolved in all patients.

In our study, 33 eyes received once-daily topical nepafenac 0.3% for at least 2 months. Median BCVA improved significantly from 0.5 to 0.3 and CRT decreased significantly from 435 to 316 μm. The resolution rate of macular edema was 57.6%.

Bellocq et al. [13] applied intravitreal dexamethasone implant in 58 eyes with PME. Of these, 14% were treatment-naïve, while the others had received previous treatments (topical NSAIDs, oral acetazolamide, intravitreal or subconjunctival triamcinolone, intravitreal anti-VEGF). The study included patients with macular edema after cataract and several other types of surgery. Twelve months after a mean of 1.7 injections, the ETDRS BCVA improved significantly from 58.5 to 71 letters (logMAR scale: approximately 0.55 to 0.3) and CRT decreased significantly from 518.3 to 342.7 μm. Intraocular pressure > 25 mmHg was observed in 6.2% of the patients, but no filtering surgery was required. The specific resolution rate of macular edema for PME at 12 months was not reported. Abdolrahimzadeh et al. [15] applied intravitreal dexamethasone implant in 10 eyes with PME after uncomplicated phacoemulsification. The patients were unresponsive to topical steroids and NSAIDs. Five patients showed recurrence after one intravitreal dexamethasone implant and they received a second implant at month 7. After a twelve-month follow-up, the ETDRS BCVA improved significantly from 62 to 79 letters and CRT decreased significantly from 622 to 282 μm. Two patients were excluded from the study, and intraocular pressure remained stable during the follow-up. Furino et al. [16] applied a single injection of intravitreal dexamethasone implant in 11 eyes with PME after uncomplicated phacoemulsification. Six months later, BCVA improved significantly from 20/40 to 20/22 and CRT decreased significantly from 462 to 276 μm. Intraocular pressure did not increase significantly during the follow-up.

Erden et al. [12] applied a single injection of 40 mg subtenon triamcinolone in 21 treatment-naïve eyes diagnosed with PME. In that study, logMAR BCVA improved significantly from 0.71 to 0.24 and CRT decreased significantly from 431 to 299 μm after 6 months. The edema resolved in 90% of patients. Although the intraocular pressure increased slightly, this increase was not statistically significant.

In our study, 13 eyes received second-line corticosteroid treatment (intravitreal dexamethasone implant or peribulbar triamcinolone) after a course of at least 2 months of topical NSAIDs. The edema resolved in 9 eyes (69.2%), with a non-significant improvement in median BCVA (from 0.7 to 0.4), and a significant decrease in CRT (from 492 to 317 μm).

In our series, macular edema resolved in 84.8% of 33 eyes treated in accordance with our treatment algorithm (Fig. 1). To our knowledge, this is the first study of PME to describe a specific treatment algorithm and the success rate. Importantly, the effectiveness of corticosteroid treatment in our series could have been underestimated because these second-line treatments were applied in recalcitrant cases that failed to respond to a previous course of topical NSAIDs. In addition, in these cases, the PME had been present for an extended period of time, and the duration of PME may hinder fluid resolution and recovery of BCVA [10, 24]. In this regard, most of the patients treated with corticosteroids showed some degree of disruption of the external retinal layers on SS-OCT, which could explain why BCVA did not improve significantly (despite a clear trend towards improvement) even though both retinal and choroidal thicknesses decreased and the edema resolved in most of these cases. Considering these results, we can hypothesize that could be a very effective first-line option for PME, but we cannot confirm this supposition based on our study.

It is worth noting that we administered triamcinolone through the peribulbar route whereas other studies have used the subtenon route [12, 23]. As a result, it is difficult to directly compare our findings in these patients with other studies. To our knowledge, these two routes of administration have not been previously compared in PME.

The effectiveness of topical NSAIDs could have been underestimated in our study given that results were evaluated after an initial two-month course of treatment (although treatment was continued if some improvement was noted). By comparison, most studies that have evaluated the effectiveness of topical NSAIDs in PME have administered a three-month course of treatment [22, 23].

To date, no randomized trials have been performed to assess the optimal therapeutic protocol for PME. However, our treatment algorithm is a common approach to the management of PME [9, 10]. Since all of the patients were treated at the same ophthalmology department, the clinical management protocol was the same in all cases, performed in accordance with common criteria. This homogeneous management approach further strengthens the internal validity of our study.

Compared to other studies, the present case series is relatively large, particularly considering the single-institutional nature of the study [11, 12, 15, 16, 18, 19].

In addition, this is the first study to evaluate choroidal thickness in PME using SS-OCT. Other studies have previously evaluated choroidal thickness in PME using spectral domain OCT (SS-OCT) [25–28]. However, to the best of our knowledge, our study is the first one to evaluate choroidal thickness in PME using Swept-Source OCT. SS-OCT devices use longer wavelengths than spectral domain ones (1050 nm versus 840 nm). As a result, they experience less light scattering on the choroid and produce more precise images of the choroid. Besides, in our study we have utilized the application of the Triton SS-OCT to define automatically the limits of the choroid, while SD-OCT devices need the operator to manually establish the limits of the choroid in every case. This fact could cause a subjective bias when using SD-OCT to measure choroidal thicknesses, while SS-OCT has proved to be highly reproducible [29–31].

Importantly, we found that CCT decreased significantly after corticosteroid treatment, a finding that appears to support the hypothesis regarding the role of inflammation in PME, which may lead to the disruption of the blood-retina barrier and thus increased vascular permeability and fluid accumulation [7, 8]. Interestingly, this decrease in choroidal thickness was not evidenced in patients treated with topical NSAIDs, potentially due to the greater effectiveness of intravitreal dexamethasone implant [22]. Although peribulbar triamcinolone can resolve PME in cases that fail to respond to topical NSAIDs, one study found no statistically significant difference between the two treatments in terms of CRT in patients with PME [23]. The changes in CCT could shed light on the pathophysiology of PME and guide new treatments and future management of PME, if the association between inflammation and PME is confirmed. The decrease in CCT that we observed in eyes treated with corticosteroids could indicate an association between PME and the pachychoroid spectrum, with choroidal thickness being elevated in PME as occurs in central serous chorioretinopathy or aneurismal type 1 neovascularization [32], but further studies are needed to verify this hypothesis.

### Study limitations

One limitation of this study is the sample size (33 eyes). Although this is larger than many of the studies on PME carried out to date, it is still insufficient to draw any definitive conclusions. Another potential limitation is the evaluation of the corticosteroid treatment, as corticosteroids were only administered in cases refractory to topical NSAIDs after several months of treatment. This could have negatively influenced the effectiveness of corticosteroid treatment in terms of resolution of the edema and final BCVA compared to upfront, first-line treatment with corticosteroids. However, this treatment sequence is widely used in ophthalmology to treat PME because NSAIDs are less invasive and have fewer side effects.

## Conclusion

In this series, topical NSAIDs were an effective treatment for PME, successfully resolving the edema in more than half of the patients. In eyes refractory to topical NSAIDs, corticosteroid treatment (intravitreal dexamethasone implant and peribulbar triamcinolone) were both effective second-line options, yielding good results in more than two-thirds of recalcitrant cases. Overall, the treatment algorithm described here was successful in nearly 85% of cases. Additional studies, preferably prospective, are needed to confirm these results.

## Data Availability

The datasets used and/or analysed during the current study are available from the corresponding author on reasonable request.
